# Editorial: The genetics of human Mendelian skin disorders

**DOI:** 10.3389/fgene.2022.1061724

**Published:** 2022-12-01

**Authors:** Wei Hsum Yap, Jia Zhang, Ming Li, Yiran Guo

**Affiliations:** ^1^ School of Biosciences, Faculty of Health and Medical Sciences, Taylor’s University, Subang Jaya, Malaysia; ^2^ Center for Drug Discovery and Molecular Pharmacology, Faculty of Health and Medical Sciences, Taylor’s University, Subang Jaya, Malaysia; ^3^ Department of Dermatology, Xinhua Hospital, Shanghai Jiaotong University School of Medicine, Shanghai, China; ^4^ Institute of Dermatology, Shanghai Jiaotong University School of Medicine, Shanghai, China; ^5^ Lineberger Comprehensive Cancer Center, School of Medicine, University of North Carolina at Chapel Hill, Chapel Hill, NC, United States; ^6^ Curriculum in Genetics and Molecular Biology, University of North Carolina at Chapel Hill, Chapel Hill, NC, United States

**Keywords:** Mendelian skin disorders, mutation, molecular mechanisms, genotype-phenotype correlations, therapy

## Introduction

The skin is comprised of multiple types of cells that serve as a protective barrier. Mutations in the genes that are responsible for protecting the functional integrity of the skin are often found in many inherited skin diseases, more commonly known as the Mendelian human skin disorders. Advances in molecular techniques and sequencing technologies have enabled identification of novel pathogenic variants, which helps to provide insight into genotype–phenotype correlations and to define the genetic basis of these skin disorders. In this Research Topic, a total of ten articles are published, including those describing findings from case studies and original research, as well as a mini review of current genetic diagnosis strategies, novel gene variants, and genotype–phenotype correlations in human Mendelian skin disorders.

### Genetic diagnosis of Mendelian skin conditions

Recent developments in genome-wide association studies (GWAS) and next-generation sequencing (NGS) techniques have resulted in an integrative approach to the use of functional genomics and expression data in deciphering the causative genetic variants of inherited skin diseases. Because most of the mutations identified in human Mendelian skin disorders reside in protein-coding genes, whole exome sequencing (WES) has been widely used in the identification of such pathogenic variants, and in the genetic diagnosis of Mendelian skin conditions with atypical or unique phenotypes. Xu et al. report on the use of WES in identifying a *de novo* pathogenic variant c.2T>C (p.M1T) in *KLHL24* in Chinese twin boys with epidermolysis bullosa simplex. Similarly, Wang et al. demonstrate the detection using WES of a missense mutation, c.1156G > A (p.Ala386Thr) in *DKC1*, which leads to a benign form of dyskeratosis congenita syndrome with the mucocutaneous triad. In another article appearing in the Research Topic, WES analysis also reveals a heterozygous missense mutation c.293G>A in *GJB3*, which is associated with erythrokeratodermia variabilis, ichthyosis, and non-syndromic hearing loss Gao et al.

Although WES is best used to characterize small indels in protein-coding exon regions, the detection of copy number variations, structural variants, and homologous regions remains challenging. Therefore, several studies have combined the use of ultra-high multiplexed PCR and ligation-dependent probe amplification techniques with WES in the form of molecular diagnostics protocols for the identification of exon variants and the development of genetic profiles of patients with human Mendelian skin disorders. This includes the detection of ectodysplasin A exon variants in X-linked hypohidrotic ectodermal dysplasia Wang et al. the genetic profiling of epidermolysis bullosa cases Alharthi et al. and the discovery of a novel CREBBP variant for genetic diagnosis of Rubinstein–Taybi Syndrome Lee et al. Recent evidence from large-scale expression studies (using microarrays and RNA sequencing) has also revealed the roles played by non-coding RNA molecules, such as small non-coding RNAs or miRNAs, in the pathogenesis of several complex skin diseases (Shefler et al., 2022). The discovery and identification of such non-coding RNAs enables them to potentially serve as diagnostic markers.

### Novel genetic variants in human Mendelian skin disorders

Molecular genetic studies based on family case reports and large-scale regional profiling analyses often provide significant insight into novel pathogenic variants, thereby helping to extend the spectrum of the genetic profile, improve diagnosis, and establish an improved understanding of human Mendelian skin disorders. In this Research Topic, Alharthi et al. report the discovery of 14 novel mutations in patients with inherited epidermolysis bullosa. This includes novel missense and frameshift mutations in gene *COL7A1* among patients with dystrophic epidermolysis bullosa and frameshift mutations in *COL17A1* and *LAMB3* among patients with junctional epidermolysis bullosa, as well as missense and non-sense mutations in genes *TGM5*, *PLEC*, and *DST* among patients with epidermolysis bullosa simplex. Meanwhile, a novel heterozygous splice-site mutation c.900-1G > C in the *ATP2C1* gene is also identified in a case report on a rare autosomal-dominant blistering disorder known as Hailey–Hailey disease Dai et al. Separately, Lee et al. have established a genetic diagnosis protocol for the detection of Rubinstein–Taybi syndrome, identifying a novel heterozygous non-sense *CREBBP* mutation (NM_004380: c. C2608T: p. Gln870Ter) with a significant pathogenicity score. In addition, a novel homozygous missense mutation (p.L154R) in gene ABHD5 has been detected in a patient with Chanarin–Dorfman syndrome, a rare autosomal recessively inherited genetic disease Liang et al. Finally, Wu et al. also report in this Research Topic on a new case of congenital poikiloderma with a novel missense mutation in the *FAM111B* gene c.1883G>A (rs587777238).

### Genotype–phenotype correlations

Certain phenotypic features of inherited skin disorders may be associated with particular gene mutations, but paradigms for clinical genotype–phenotype correlation remain unclear in many instances due to the highly variable phenotypic expressivity of the relevant mutations; these paradigms require further refinement. In this Research Topic, Xu et al. provide an initial description of their discovery of a c.2T>C pathogenic variant in *KLHL24*, affecting twins in China, and its correlation with epidermolysis bullosa simplex. Their research has identified correlations between phenotypes and genotypes in epidermolysis bullosa, in which *KLHL24* pathogenic variants are associated with the mild phenotype. In contrast, the study by Liang et al. on genotype–phenotype analysis in patients with reported Chanarin–Dorfman syndrome reveals no correlation. Meanwhile, as indicated in a review by How et al. there is no significant genotype–phenotype relation in incontinentia pigmenti, a rare type of X-linked dominant genetic disease characterized by ectodermal dysplastic disorder. However, the literature does suggest that variation in a combination of the types of mutations, functional domains affected, X-inactivation, and genomic background may lead to the variability observed in incontinentia pigmenti phenotypes How et al. The authors propose that a detailed understanding of the genotype–phenotype correlation in incontinentia pigmenti will support further investigations concerning prognosis and future reproductive options. Meanwhile, a recent large cohort study involving investigation of genotype–phenotype correlations in patients with autosomal recessive ichthyosis has provided new insights on and definitions of specific phenotypic clues for corresponding genetic mutations (Simpson et al., 2020). In addition to the need for large cohort trials, some researchers have proposed the use of computational approaches to connecting patient phenotypes based on phenotypic co-occurrence, combined with the use of genomic information related to the mutations found in each patient, to correlate genes with phenotypes; this type of approach can be used to investigate the relevant functional systems (Díaz-Santiago et al., 2020).

## Conclusion

In summary, this Research Topic enhances our knowledge of recent exciting progress in the field of genodermatosis, including molecular diagnostics protocols, novel pathogenetic variants, and genotype–phenotype correlations. Together, these studies provide value in the form of greater diagnostic precision, a source of information for clinical assessments, and ways to improve clinical care and management.

## References

[B1] Díaz-Santiago E. Jabato F. M. Rojano E. Seoane P. Pazos F. Perkins J. R. (2020). Phenotype-genotype comorbidity analysis of patients with rare disorders provides insight into their pathological and molecular bases. PLoS Genet. 16 (10), e1009054.. 10.1371/journal.pgen.1009054 33001999 PMC7553355

[B2] Shefler A. Patrick M. T. Wasikowski R. Chen J. Sarkar M. K. Gudjonsson J. E. (2022). Skin-expressing lncRNAs in inflammatory responses. Front. Genet. 13, 835740. 10.3389/fgene.2022.835740 35559048 PMC9086234

[B3] Simpson J. K. Martinez-Queipo M. Onoufriadis A. Tso S. Glass E. Liu L.. (2020). Genotype-phenotype correlation in a large English cohort of patients with autosomal recessive ichthyosis. Br. J. Dermatol. 182 (3), 729–737. 10.1111/bjd.18211 31168818

